# Wave Intensity Analysis Combined With Machine Learning can Detect Impaired Stroke Volume in Simulations of Heart Failure

**DOI:** 10.3389/fbioe.2021.737055

**Published:** 2021-12-24

**Authors:** Ryan M. Reavette, Spencer J. Sherwin, Meng-Xing Tang, Peter D. Weinberg

**Affiliations:** ^1^ Department of Bioengineering, Imperial College London, London, United Kingdom; ^2^ Department of Aeronautics, Imperial College London, London, United Kingdom

**Keywords:** wave intensity analysis, pulse waves, 1D arterial haemodynamics, machine learning, heart failure

## Abstract

Heart failure is treatable, but in the United Kingdom, the 1-, 5- and 10-year mortality rates are 24.1, 54.5 and 75.5%, respectively. The poor prognosis reflects, in part, the lack of specific, simple and affordable diagnostic techniques; the disease is often advanced by the time a diagnosis is made. Previous studies have demonstrated that certain metrics derived from pressure–velocity-based wave intensity analysis are significantly altered in the presence of impaired heart performance when averaged over groups, but to date, no study has examined the diagnostic potential of wave intensity on an individual basis, and, additionally, the pressure waveform can only be obtained accurately using invasive methods, which has inhibited clinical adoption. Here, we investigate whether a new form of wave intensity based on noninvasive measurements of arterial diameter and velocity can detect impaired heart performance in an individual. To do so, we have generated a virtual population of two-thousand elderly subjects, modelling half as healthy controls and half with an impaired stroke volume. All metrics derived from the diameter–velocity-based wave intensity waveforms in the carotid, brachial and radial arteries showed significant crossover between groups—no one metric in any artery could reliably indicate whether a subject’s stroke volume was normal or impaired. However, after applying machine learning to the metrics, we found that a support vector classifier could simultaneously achieve up to 99% recall and 95% precision. We conclude that noninvasive wave intensity analysis has significant potential to improve heart failure screening and diagnosis.

## Introduction

Heart failure (HF) is a broad spectrum of disease in which the heart is unable to supply blood at the rate required by the body. It is often stratified by ejection fraction (EF): heart failure with reduced ejection fraction (≤40%, HFrEF), where there is usually a decrease in stroke volume (SV) due to a failure of intrinsic inotropy or loss of functional heart muscle; and heart failure with preserved ejection fraction (≥50%, HFpEF), where there is often a decrease in SV because the end diastolic volume (EDV) has reduced due to loss of ventricular compliance.

HF is treatable but the prognosis is poor: in the UK, the 1-, 5- and 10-year mortality rates are 24.1, 54.5 and 75.5%, respectively, and these have only improved by around 7% each since the year 2000 (Taylor et al., 2019). Moreover, 79% of UK HF diagnoses are only made after an emergency hospital admission despite 41% of the patients visiting their GP in the preceding five years with at least one of the three major symptoms of HF: breathlessness, ankle swelling, and fatigue (Bottle et al., 2018). The survival rates following a diagnosis in primary care are, as expected, better than the average (Taylor et al., 2017), but there are clear missed opportunities for early diagnosis and intervention. There is also a significant disconnect between clinical guidelines and actual patient diagnoses: only 24% of patients were diagnosed according to the recommended pathway (Taylor et al., 2019). All of these factors necessitate an improved diagnosis pipeline.

One prominent issue is that the three main symptoms are not exclusive to HF. If one of these symptoms is identified, the current pathway recommends a blood test for brain natriuretic peptides (BNP); this is highly sensitive and can therefore effectively rule out those who do not have the disease, but BNP levels are also commonly elevated in other pathologies such as chronic kidney disease (Maisel et al., 2008; Tagore et al., 2008) and may be abnormally low in obese patients (Madamanchi et al., 2014). A positive BNP test should be followed by echocardiography, which is the gold-standard for diagnosis, but this is not available in primary care and, at least in the UK, a shortage of trained staff is a limiting factor with hospital wait times of up to 6 weeks (Cowie, 2017). Even for those patients who have a HF symptom recorded during a primary care consultation, 44% are not referred for a BNP test, for echocardiography, or to a specialist for further consultation, highlighting a lack of both confidence in current investigations and test availability (Bottle et al., 2018). There is an urgent need for a specific, low-cost, noninvasive method to improve HF screening in primary care and for treatment management at point-of-care.

Arterial pulse waves carry information about the performance of the heart and vessels, and several studies have identified statistically significant changes in the metrics derived from wave intensity (WI) in the presence of impaired heart performance (Curtis et al., 2007; Siniawski et al., 2009; Li and Guo, 2013; Takaya et al., 2013; Vriz et al., 2015) but only when averaged over groups. Furthermore, WI has generally been determined from measurements of blood velocity and pressure made throughout the cardiac cycle (Parker, 2009), but pressure waveforms with sufficient temporal resolution can only be obtained accurately by invasive methods or estimated inaccurately by noninvasive ones, which has inhibited the clinical realisability of WI-based diagnostic methods.

A new form of WI has recently been introduced that instead relies upon measurements of blood velocity and arterial diameter (Feng and Khir, 2010); this is significant because both can be noninvasively measured at the same arterial location and time, for example by ultrasound. Despite the intrinsic nonlinear relationship between arterial diameter and blood pressure arising from effects such as viscoelasticity and strain-stiffening, Reavette et al. (2020) found by numerical modelling that this noninvasive method gave results that were close to those of the invasive method.

As noted above, impaired SV is important in both HFrEF and HFpEF (A reduced SV is consistent with a preserved ejection fraction if EDV is reduced.) HF can co-exist with a normal resting SV if the heart compensates with increased inotropy; however, the hearts of such patients will struggle to fulfil physiological needs during exertion. At such times, SV can again become subnormal.

To elucidate whether diameter-based WI can detect impaired heart performance on an individual basis, we have generated an age-stratified virtual population of elderly subjects, modelling half as healthy controls and half as HF patients with an impaired SV. Simulations were performed using the PulseWaveSolver utility of Nektar++ (Cantwell et al., 2015), which solves the 1D equations of blood flow using a high-order discontinuous Galerkin method with a spectral/hp-element discretisation. This reduced-order modelling can accurately solve for the arterial area, velocity and pressure waveforms in complex arterial networks with reasonable computational cost (Alastruey et al., 2011; Sherwin et al., 2003) and has been validated against *in-vitro* (Alastruey et al., 2011; Matthys et al., 2007) and *in-vivo* (Mynard and Smolich, 2015; Olufsen et al., 2000; Pedregosa et al., 2011) data. We applied a support vector machine (SVM) classifier to metrics derived from wave intensity analysis (WIA) in the common carotid, brachial and radial arteries. These arteries are all accessible to ultrasound. Machine learning can identify and utilise complex relationships between metrics and has been successfully applied in numerous biomedical classification studies (Yamamoto et al., 2020; Zhao et al., 2020) and to *in-silico* datasets (Bikia et al., 2021; Jin et al., 2021). An SVM was chosen after it performed best in preliminary tests against other classification algorithms.

## Materials and Methods

**FIGURE 1:**
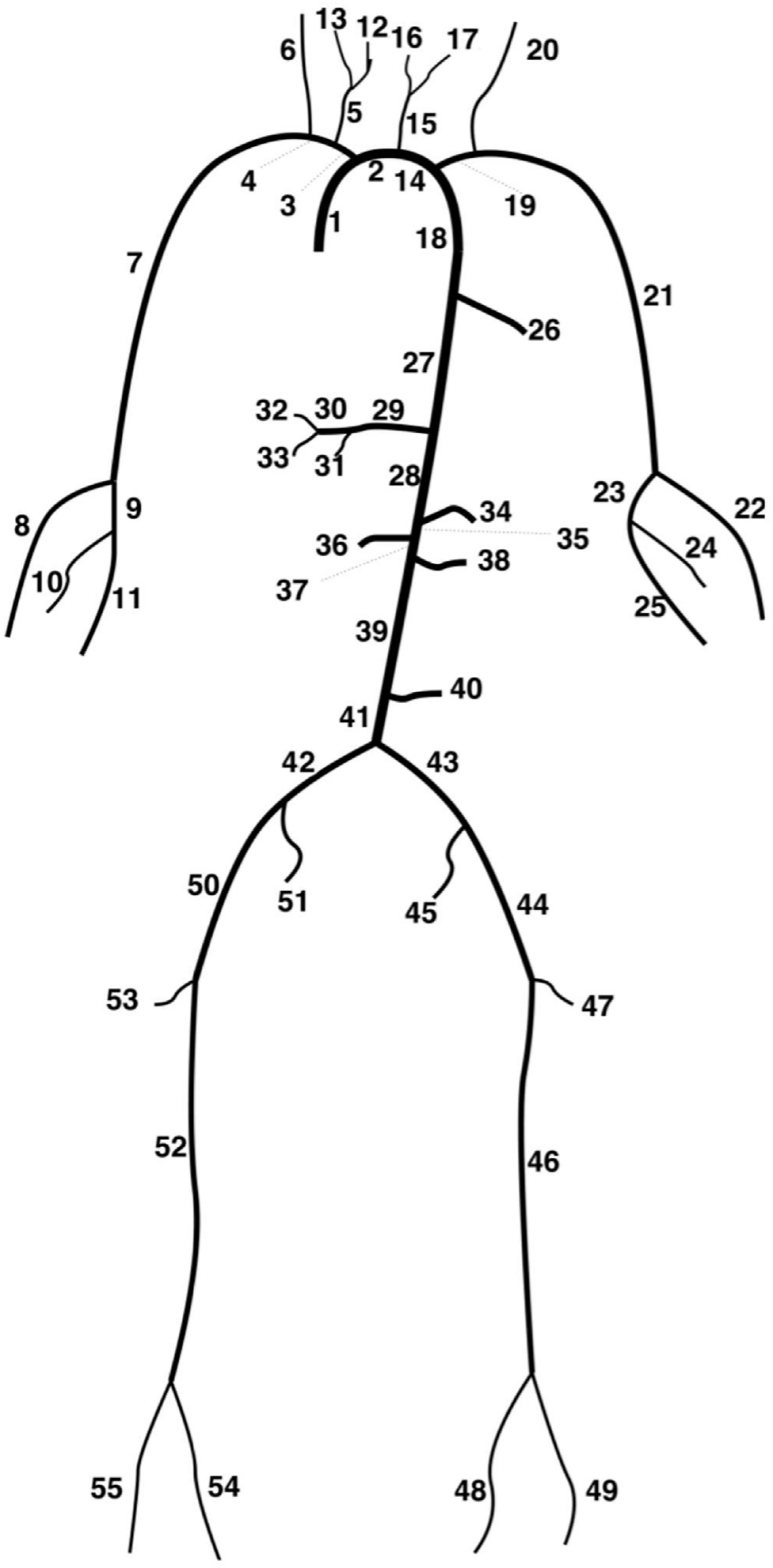
Depiction of the 55-artery network (Willemet, 2015a).

The 1D equations of conservation of mass and momentum

$$\frac{\partial A}{\partial t}+\frac{\partial }{\partial x}\left(AU\right)=0,$$ (1)

$$\frac{\partial U}{\partial t}+U\frac{\partial U}{\partial x}+\frac{1}{\rho }\frac{\partial P}{\partial x}=\frac{f}{\rho A},$$ (2)

for cross-sectional area A, average cross-sectional velocity U and pressure P, frictional force per unit length f, and density of blood ρ = 1,060 kg m^−3^, together with the nonlinear tube law

$$P=P_{d}-\frac{\beta \sqrt{A}}{2\alpha }\ln\left[1-\alpha \ln\left(\;\frac{A}{A_{d}}\;\right)\right]+\frac{\Gamma }{\sqrt{A}}\frac{\partial A}{\partial t},$$ (3)

for reference diastolic area A_d_ and pressure P_d_, stiffness and viscoelastic parameters β and Γ , respectively, and strain-stiffening modulus α , were solved in models of the largest 55 systemic arteries (Figure 1). The arteries were modelled as linearly tapering vessels, and terminal vessels were coupled to RCR Windkessel models (Alastruey et al., 2012). For further details of the numerical method and tube law, the reader is referred to Sherwin et al. (2003) and Reavette et al. (2020), respectively. The simulations and analysis were performed locally on an iMac (2017, 4.2 GHz Quad-Core Intel Core i7 processor, 32 GB RAM) using Nektar++ (Cantwell et al., 2015) and Python (Van Rossum and Drake, 2009), respectively.

### Generation of a Virtual Population

Two-thousand subjects were generated, with equal numbers of males and females, of controls and HF patients, and in age groups of 60–69 and 70–79 years. To simulate blood flow in each subject, it is necessary to specify the parameters of the arterial network—such as arterial areas, lengths and wave speeds—and an inflow waveform.

To generate network parameters for each subject, we took those from the model of Willemet et al. (2015b, Table 1), with wall viscosities from Alastruey et al. (2012), and scaled them using randomly generated age-stratified multipliers in the physiological ranges given in Table 2, which is based on similar tables given in Reavette et al. (2020) and Willemet et al. (2015b). Arterial lengths were scaled to account for men being 7% taller than women (Roser et al., 2019). Furthermore, for all groups the total peripheral resistance R, total peripheral compliance C, arterial lengths L_i_, strain-stiffening parameters α_i_, wall viscosities ϕ_i_, diastolic pressure P_d_ and outflow pressure to the venous system P_out_ were varied with multipliers of 0.9–1.1. Doing so provided additional variation between subjects without inducing significant deviation from published values.

**TABLE 1:** Parameters of the baseline model. In addition to these, P_d_ and P_out_ were 10 kPa (75 mmHg) and 1.33 kPa (10 mmHg), respectively.

Artery	Length, *L* (cm)	Prescribed area, *A*_d, in_ *→* *A*_d, out_ (cm^2^)	Wave speed Coefficient, *a*_*c*_	Wall viscosity, *ϕ* (kg cm^−1^s^−1^)	Peripheral resistance, *R* (kg cm^−4^s^−1^)	Peripheral compliance, *C* (cm^4^s kg^−1^)
1. Ascending aorta	5.8	7.21 *→* 7.16	14.3	5	-	-
2. Aortic arch A	2.3	5.23 *→* 4.79	14.3	5	-	-
3. Brachiocephalic	3.9	3.40 *→* 2.69	14.3	10	-	-
4. R. subclavian	3.9	1.09 *→* 0.675	14.3	10	-	-
5. R. common carotid	10.8	1.00 *→* 0.270	14.3	60	-	-
6. R. vertebral	17.1	0.114 *→* 0.0651	15.6	60	45.1	0.00902
7. R. brachial	48.5	0.556 *→* 0.184	15.6	25	-	-
8. R. radial	27.0	0.114 *→* 0.0799	15.6	60	39.6	0.00987
9. R. ulnar A	7.7	0.114 *→* 0.0962	15.6	60	-	-
10. R. interosseous	9.1	0.0366 *→* 0.0269	15.6	60	632	0.00325
11. R. ulnar B	19.7	0.0850 *→* 0.0651	15.6	60	39.6	0.00769
12. R. internal carotid	20.5	0.271 *→* 0.153	15.6	60	18.8	0.0258
13. R. external carotid	18.7	0.0519 *→* 0.0186	15.6	60	104	0.0193
14. Aortic arch B	4.5	3.80 *→* 3.60	14.3	5	-	-
15. L. common carotid	16.0	0.785 *→* 0.198	14.3	60	-	-
16. L. internal carotid	20.5	0.154 *→* 0.0924	15.6	60	18.8	0.0189
17. L. external carotid	18.7	0.0305 *→* 0.0125	15.6	60	104	0.0173
18. Thoracic aorta A	6.0	3.33 *→* 2.99	14.3	5	-	-
19. L. subclavian	3.9	1.00 *→* 0.590	14.3	10	-	-
20. L. vertebral	17.0	0.114 *→* 0.0651	15.6	60	45.1	0.00902
21. L. brachial	48.5	0.546 *→* 0.184	15.6	25	-	-
22. L. radial	27.0	0.102 *→* 0.0651	15.6	60	39.6	0.00848
23. L. ulnar A	7.7	0.154 *→* 0.154	15.6	60	-	-
24. L. interosseous	9.1	0.0269 *→* 0.0269	15.6	60	632	0.00277
25. L. ulnar B	19.7	0.140 *→* 0.114	15.6	60	39.6	0.0130
26. Intercostals	9.2	1.33 *→* 0.751	14.3	5	60.0	0.104
27. Thoracic aorta B	12.0	2.27 *→* 1.39	14.3	5	-	-
28. Abdominal aorta A	6.1	1.25 *→* 1.25	14.3	5	-	-
29. Celiac A	2.3	0.506 *→* 0.395	14.3	5	-	-
30. Celiac B	2.3	0.225 *→* 0.200	14.3	5	-	-
31. Hepatic	7.6	0.243 *→* 0.161	15.6	25	27.2	0.0205
32. Gastric	8.2	0.0850 *→* 0.0750	15.6	60	40.6	0.00821
33. Splenic	7.2	0.147 *→* 0.127	15.6	60	17.4	0.0140
34. Superior mesenteric	6.8	0.519 *→* 0.420	14.3	10	6.98	0.0481
35. Abdominal aorta B	2.3	1.09 *→* 1.06	14.3	5	-	-
36. L. renal	3.7	0.225 *→* 0.225	14.3	25	8.48	0.0231
37. Abdominal aorta C	2.3	1.15 *→* 1.15	14.3	5	-	-
38. R. renal	3.7	0.225 *→* 0.225	14.3	25	8.48	0.0231
39. Abdominal aorta D	12.2	1.11 *→* 1.00	14.3	5	-	-
40. Inferior mesenteric	5.8	0.184 *→* 0.0844	15.6	25	51.6	0.0133
41. Abdominal aorta E	2.3	0.968 *→* 0.899	14.3	5	-	-
42. L. common iliac	6.8	0.519 *→* 0.407	18.0	10	-	-
43. R. common iliac	6.8	0.519 *→* 0.407	18.0	10	-	-
44. L. external iliac	16.6	0.341 *→* 0.310	18.0	25	-	-
45. R. internal iliac	5.8	0.133 *→* 0.133	19.7	60	59.6	0.0137
46. L. femoral	50.9	0.225 *→* 0.120	19.7	60	-	-
47. L. deep femoral	14.5	0.133 *→* 0.114	19.7	60	35.8	0.0127
48. L. posterior tibial	36.9	0.0799 *→* 0.0651	19.7	60	106	0.00743
49. L. anterior tibial	39.8	0.0564 *→* 0.0441	19.7	60	106	0.00513
50. R. external iliac	16.6	0.341 *→* 0.310	18.0	25	-	-
51. R. internal iliac	5.8	0.133 *→* 0.133	19.7	60	59.6	0.00137
52. R. femoral	50.9	0.225 *→* 0.120	19.7	60	-	-
53. R. deep femoral	14.5	0.133 *→* 0.114	19.7	60	35.8	0.0127
54. R. posterior tibial	36.9	0.0799 *→* 0.0651	19.7	60	106	0.00743
55. R. anterior tibial	39.8	0.0564 *→* 0.0441	19.7	60	106	0.00513

**TABLE 2:** Variation for the parameters that were known to vary between age groups. Arteries were categorised based on structure: 1–5, 14, 15, 18, 19, 26–30, 35–39 and 41 as elastic and the rest muscular. PWV = Pulse wave velocity.

Parameter	Multiplier	
	Age group (year)	
	60–69	70–79
Elastic arteries PWV (*c*_elas_)	1.75–2.05	2.075–2.425
Muscular arteries PWV (*c*_musc_)	1.075–1.225	1.225–1.375
Elastic arteries diameter (*D*_elas_)	1.1–1.3	1.3–1.5
Muscular arteries diameter (*D*_musc_)	1.105–1.305	

To generate a different inflow waveform for each subject, we took that used for the thoracic aorta by Boileau et al. (2015), added natural variation to its shape, and scaled it to match a generated SV, heart rate (HR) and left ventricular ejection time (LVET).

To generate an SV for each subject, we started with the 2D echocardiographic values for EDV and EF given in Table 3 (Lang et al., 2005; Lang et al., 2015). These were assumed to be normally distributed, and values for each subject were randomly sampled from each distribution (McDonagh et al., 2021). For each control, the EDV and EF were multiplied to calculate an SV, and this was used if it was in the range 60–100 ml (Edwards Lifesciences LLC, 2009). For each patient, the EDV was arbitrarily multiplied by 0.9 to reduce it, and the resulting SV was used if it was below 60 ml. Echocardiographic values were used for the EDV and EF ranges because they are generally used to define EFs clinically. They are lower than those obtained using MRI (Maceira et al., 2006).

**TABLE 3:** Typical echocardiographic parameters (Lang et al., 2005; Lang et al., 2015). SD = Standard deviation.

	Male (Mean ± SD)	Female (Mean ± SD)
End Diastolic Volume (ml)	106 ± 22	76 ± 15
Ejection Fraction (%)	62 ± 5	64 ± 5

**FIGURE 2:**
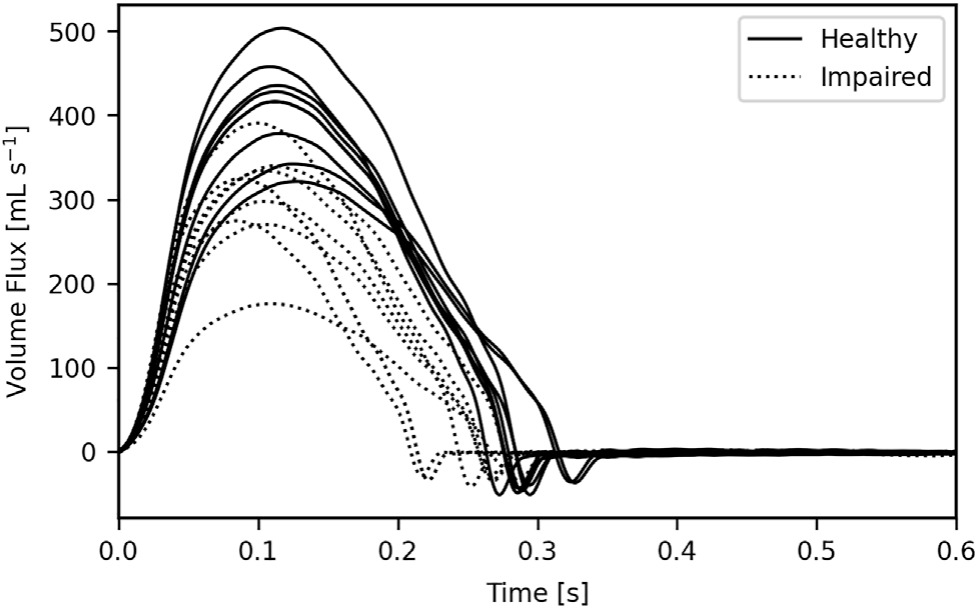
16 example inflow waveforms. The area under each waveform gives the respective stroke volume; this was impaired through either a shortened left ventricular ejection time, a reduced peak inflow, or a combination of the two.

An HR in the range 60–90 bpm was randomly assigned to each subject and used to calculate an LVET through regression analysis of the data given in Weissler et al. (1968) with natural variation added to the LVET. Example waveforms are given in Figure 2.

The average SV for women is lower than for men. To reflect this difference, the cut-off of 60 ml between healthy and impaired subjects should theoretically be lower for women. Keeping the same cut-off for both sexes, however, allowed us to test the algorithms on two distinct SV distributions: for men, SVs would be more dispersed, with greater average differences between controls and HF patients; for women, the SVs would be more concentrated around the 60 ml mark for both the controls and HF patients. We were interested in measuring how the classifier performed in each case.

The cardiac output for each control was restricted to a healthy physiological range of 4–8 L min^−1^. Both normal and reduced cardiac outputs were permitted for HF patients to account for those whose compensatory mechanisms are and are not working, respectively (Brandforbrener et al., 1955; Curtis et al., 2007; Rodeheffer et al., 1984; Vriz et al., 2015).

Resistances were scaled to bring brachial blood pressures into the physiological range: diastolic blood pressures were normal, with a mean of 74 mmHg across all subjects; systolic blood pressures were often elevated, with a mean of 119 mmHg across all subjects and with numerous subjects exhibiting isolated systolic hypertension from the increased arterial stiffness observed in the elderly. The resistances of HF patients were scaled by a larger amount to reflect the physiological compensatory mechanism triggered by a reduced cardiac output; this should introduce distinctions in the reflection coefficients between the controls and HF patients.

### Filter Criteria

Following Willemet et al. (2015b), the SV and cardiac output filter criteria were supplemented by additional restrictions based on brachial blood pressure and the aorto-iliac bifurcation reflection coefficient (Willemet et al., 2016), which was calculated as

$$R_{a}=\frac{Y^{a}-Y^{b}-Y^{c}}{Y^{a}+Y^{b}+Y^{c}},Y^{i}=\frac{\rho c_{i}}{A_{i}}.$$ (4)

All criteria are summarised in Table 4; whenever subjects did not pass all filters, replacements were generated.

**TABLE 4:** Filter criteria. Blood pressures refer to those taken in the brachial artery, and the reflection coefficient was calculated at the aorto-iliac bifurcation using Eq. 4 (Willemet et al., 2015b).

Criterion	Range	Group
Stroke Volume	60–100 ml	Controls
	<60 ml	Patients
Cardiac Output	4–8 L/min	Controls
Diastolic Blood Pressure	*≥*40 mmHg	All
Systolic Blood Pressure	≤200 mmHg	All
Pulse Pressure, *P*_pulse_	25 mmHg ≤ P_pulse_ ≤ 100 mmHg	All
Reflection Coefficient, *R*_*a*_	*−*0.3 ≤ *R*_*a*_ ≤ 0.3	All

### Wave Intensity

Diameter-based WI was calculated as

$$dI=\frac{dDdU}{{\left(\Delta t\right)}^{2}},$$ (5)

where the scaling by ${\left(\Delta t\right)}^{2}$ removes the dependence of the magnitude on the sampling period.

WI is separable into intensities of forwards- and backwards-travelling waves,

$$dI_{\pm }=\pm \frac{1}{{\left(\Delta t\right)}^{2}}\frac{c}{2D}{\left(dD\pm \frac{D}{2c}dU\right)}^{2},$$ (6)

but this requires the pulse wave velocity (PWV), c ; in clinical practice, this can be estimated noninvasively through the lnDU -loop (Feng and Khir, 2010), but this introduces errors, particularly when close to significant reflection sites as in the case of the carotid artery (Willemet et al., 2016). We applied the SVM to the WI metrics generated from both the unseparated and separated WI, where for the latter we used the PWV calculated through the lnDU -loop.

### Metrics

**FIGURE 3:**
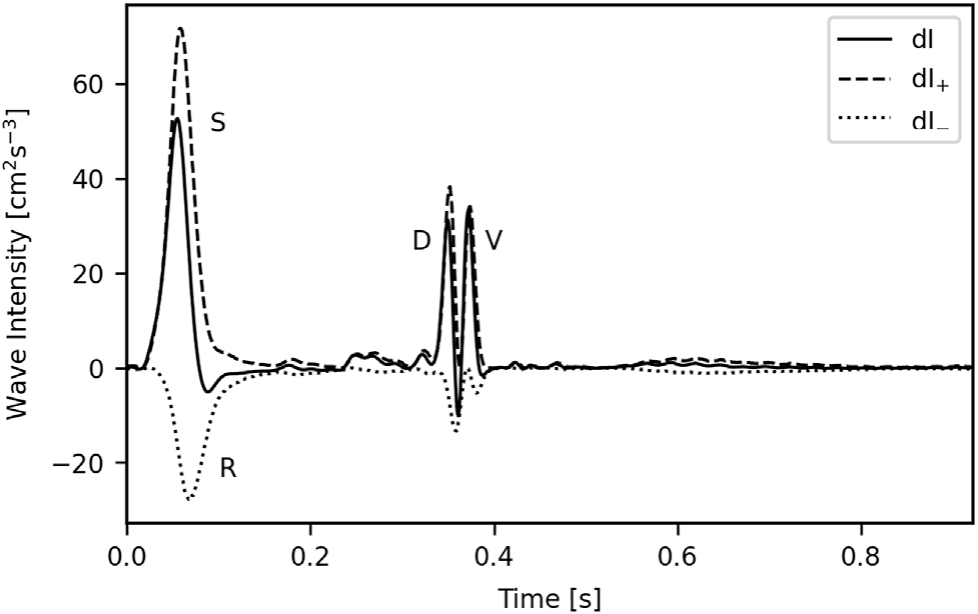
A typical wave intensity plot for the common carotid. The wave to the right of the diastolic (D) wave, the valve (V) wave, results from the abrupt closure of the aortic valve and is in turn responsible for the incisura; this is unlikely to have any diagnostic utility so was not included in the analysis. S = Systolic wave; R = Reflected wave.

A typical WI waveform for the common carotid, with separated waves, is given in Figure 3.

Eight metrics were used to characterise the waves: the magnitudes of the systolic, diastolic and reflected (S, D, and R) waves (SWI, DWI and RWI, respectively); the wave energies of the S, D, and R waves (SWE, DWE and RWE, respectively), calculated as the area under each peak; the reflection coefficient (RC), calculated as RWI/SWI; and the SD-Delay, which is the time delay between the arrivals of the peaks of the S and D waves.

### Classification

The data were organised into training and test sets using an 80/20 split—giving 1,600 and 400 subjects in the respective groups. Such a split ensured sufficient data for full training (explored in Section 3.2.1) whilst leaving enough unseen data for evaluation. An SVM with a radial basis function kernel was implemented using Scikit-learn (Cortes and Vapnik, 1995; Pedregosa et al., 2011); this kernel supports nonlinear classification. The model’s hyperparameters were optimised using 10-fold cross-validation (CV): the training set was split into ten distinct folds, and for each set of candidate hyperparameters, the model was trained ten times—each time a different fold was held out for validation with the model trained on the remaining nine. The model was then trained with the hyperparameters that had the best average performance on the validation sets, giving a model capable of generalising well to the test set.

The performance of the SVM was evaluated using the precision, recall and F1 score:

$$\text{Precision}=\frac{\text{True}\;\text{Positives}}{\text{True}\;\text{Positives}+\text{False}\;\text{Positives}}$$ (7)

$$\text{Recall}=\frac{\text{True}\;\text{Positives}}{\text{True}\;\text{Positives}+\text{False}\;\text{Negatives}}$$ (8)

$$F_{1}\;\text{Score}=2\times \frac{\text{Precision}\times \text{Recall}}{\text{Precision}+\text{Recall}}$$ (9)

Precision quantifies how many of the subjects that the model identifies as having HF actually have HF; recall quantifies how many of the HF patients the model identifies as having HF; the F_1_ score is high only when both precision and recall are high. The classifier was considered fully trained when the F_1_ score of the validation set stopped improving.

The decision threshold of the SVM can be shifted from its default value of zero to achieve the desired precision or recall, although as one increases the other decreases. Contextually, it is better to prioritise recall: more patients who show signs of HF will be detected, and those who are incorrectly identified can be ruled out by subsequent echocardiogram. Therefore, for each artery, we have included results for both the default decision threshold and that which achieved 99% recall on the training set.

The performance of the SVM on the test sets was also evaluated using confusion matrices, which show the number of true and false predictions (Table 5).

**TABLE 5:** The structure of a confusion matrix.

		Actual	
		1	0
Predicted	1	True Positive	False Positive
	0	False Negative	True Negative

## Results

Separating waves into their forwards and backwards components did not improve the model’s performance, nor did stratifying the data by age or sex; we therefore only present the results obtained from the unseparated waves for the entire dataset.

**FIGURE 4:**
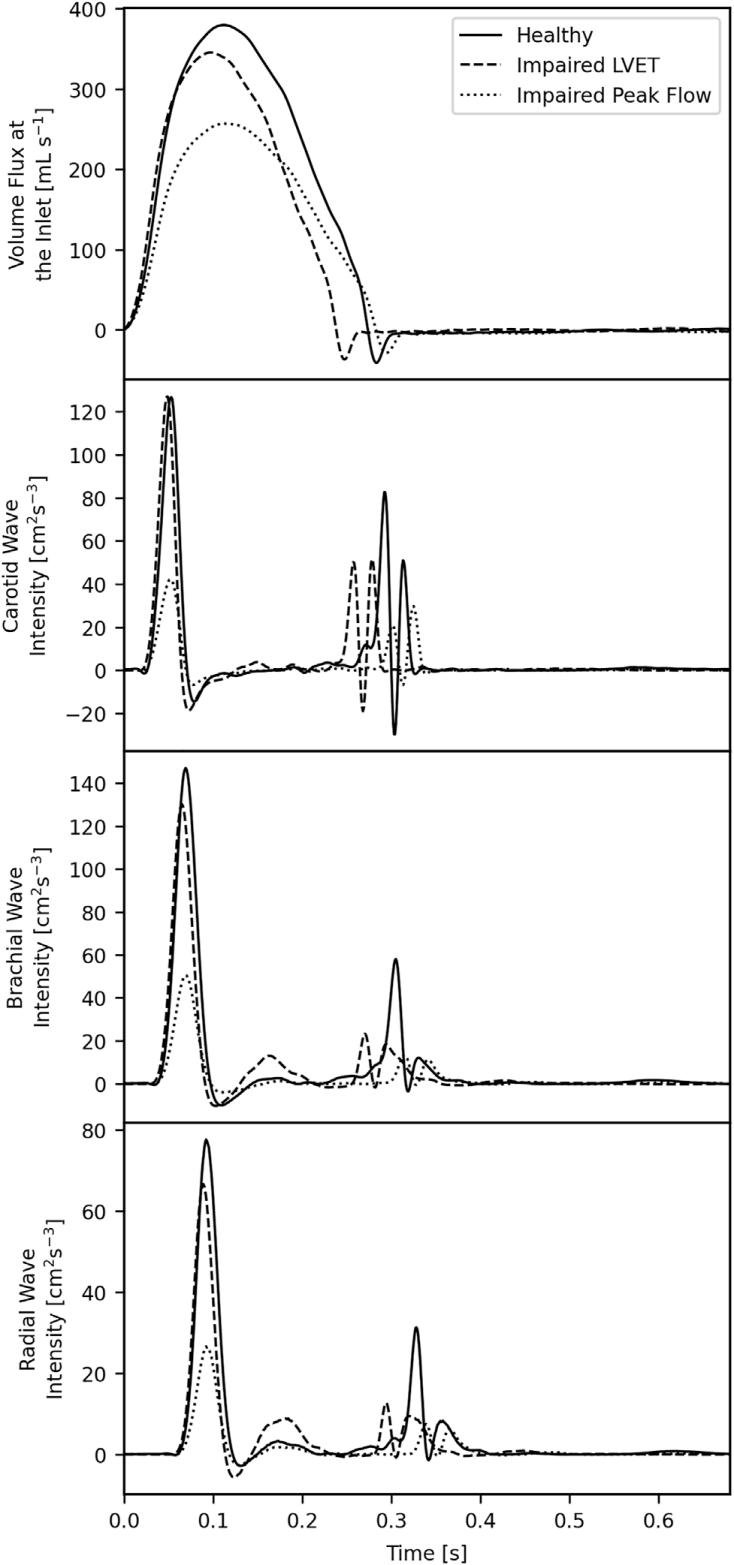
Inlet waveforms **(top)** and wave intensities in the common carotid **(second from the top)**, brachial **(second from the bottom)** and radial **(bottom)** for three subjects: one healthy control, one with an impaired left ventricular ejection time, and one with an impaired peak flow.

WI plots for the common carotid, brachial and radial arteries for three example subjects—one healthy control, one HF patient with an impaired LVET, and one HF patient with an impaired peak flow—are given in Figure 4.

The SWI and SWE are greatly reduced in all arteries for the patient with an impaired peak flow, but these same metrics are largely unchanged in the patient with impaired LVET. The DWI and DWE are reduced in all arteries for both HF patients, implying that these metrics have the potential to be the two biggest discriminators between the controls and patients. The SD-Delay is shorter for the patient with impaired LVET, as the diastolic relaxation occurs earlier due to the impairment. The R wave is approximately the same in the control and patient with an impaired LVET but is reduced in the patient with an impaired peak flow.

### Correlations

**FIGURE 5:**
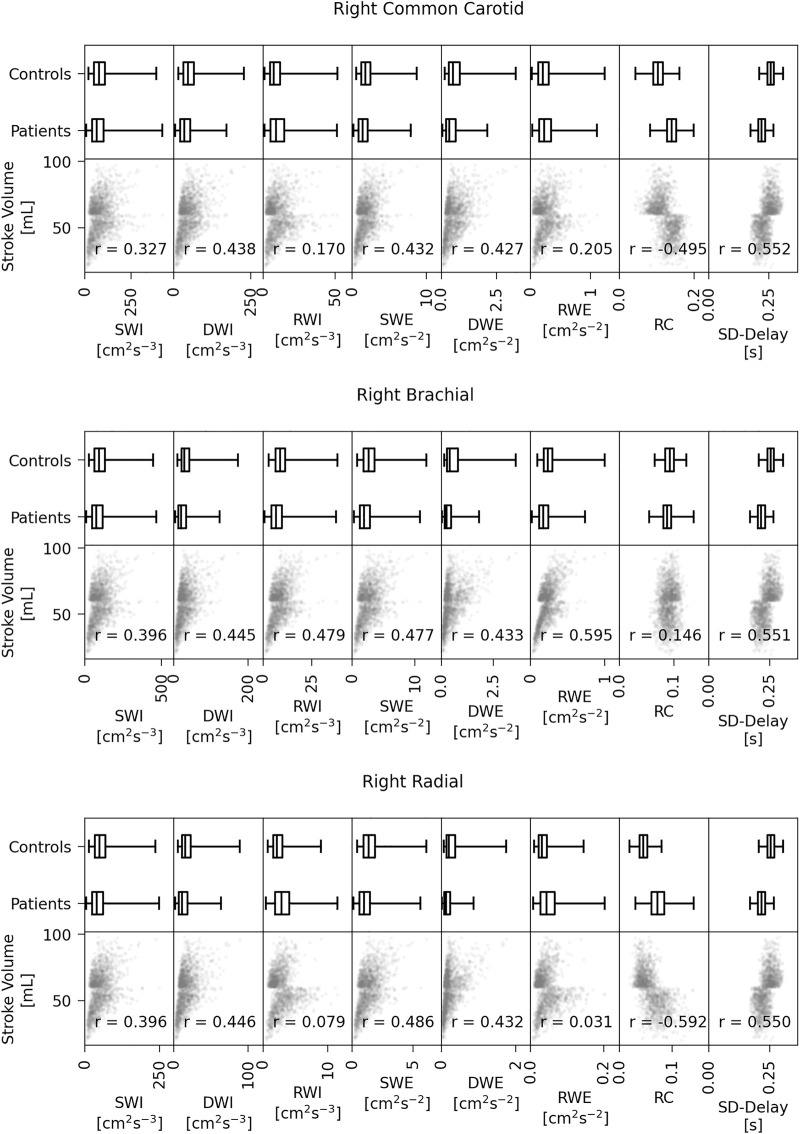
Box plots of the metric distributions separated by controls and patients (top row of each) and the stroke volume against all eight metrics for all three arteries for the training set data (bottom row of each). SWI, RWI and DWI = S, R and D wave intensities; SWE, RWE and DWE = S, R, and D wave energies; RC = Reflection coefficient; SD-Delay = The time delay between the arrivals of the S and D waves.

Plots of SV against the various WI metrics for all arteries and subjects in the training set, as well as box plots showing the distribution of each metric stratified by controls and patients, are given in Figure 5. For each metric we have calculated the Pearson’s correlation coefficient between the metric and the SV. The arbitrary hard cut-off for the SV of 60 ml leads to a distinct horizontal line in each correlation graph, an artefact that is a consequence of generating the two groups from different distributions.

For the common carotid, all metrics except for the RWI and RWE showed moderate correlation (magnitude above 0.3) with the SV. Furthermore, although there is crossover between the metric values in the control group and those in the patient group, specific values of some metrics occurred only in one group; for example, only in the control group were DWI and DWE values ever above 175 cm^2^ s^−3^ and 2.1 cm^2^ s^−2^, respectively. The reflection coefficient increases with decreasing SV, meaning that, generally, HF patients have a higher RWI relative to the SWI, which is consistent with the result of Curtis et al., 2007.

For the brachial, the highest correlation coefficient is that of the RWE (0.595), and with the exception of the RC, all metrics showed moderate correlation with SV. Again, certain values of some of the metrics (the DWI, DWE, RWE and SD-Delay) occurred only in the control group. The RC is unlikely to influence the classification.

For the radial, the correlation coefficient with the largest magnitude is that of the RC (0.592). As with the carotid, the RC increases with decreasing SV. All metrics except for the RWI and RWE showed moderate correlation.

Although there appears to be some stratification between the two groups, no one metric in any artery can reliably be used to classify a subject’s SV as either normal or impaired. However, the correlations give only an introductory insight, as not all decreases in SV will cause the same changes in the metrics; it will depend on how the decrease arises. For example, an impaired peak flow is likely to affect the SWI and SWE, whereas an impaired LVET is likely to affect the SD-Delay.

### Classification

#### Training

**FIGURE 6:**
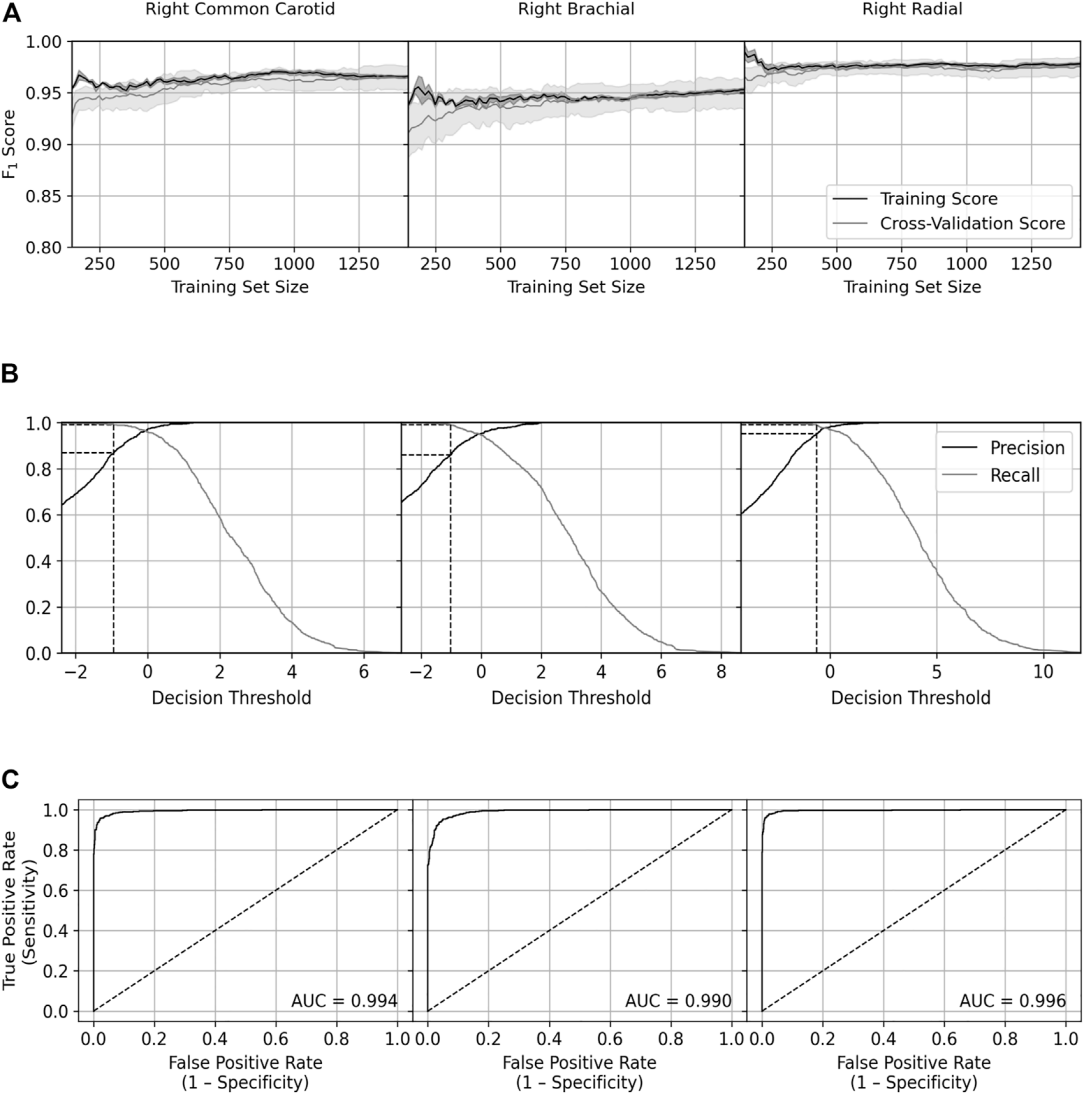
Plots of the **(A)** learning curve, **(B)** precision and recall against the decision threshold, and **(C)** receiver operating characteristic curve for each artery, all for when the classifier was applied to training data. For **(A)** the scores were the average of all nine- and one-fold training and validation sets, respectively, and the shading represents the standard deviation. For **(B)** the dashed lines show the precision at 99% recall. For **(C)** the dashed line shows the result of a perfectly random classifier. AUC = Area under the curve.

Plots of the learning curves for the training and CV sets, precision and recall against the decision threshold for the training set and the receiver operating characteristic (ROC) curve for the training set are given for each artery in Figure 6, and summary statistics are given in Table 6.

**TABLE 6:** Summary statistics for classifier training performance. ROC AUC = Area under the receiver operating characteristic curve; CV = Cross validation.

	**Training set size for full training**	**CV set final F_1_ score**	**Training set size for 97.5% of final CV F_1_ score**	**Precision at 99% recall**	**ROC AUC**
Common Carotid	1,200–1,300	0.965	170	0.868	0.994
Brachial	1,250–1,350	0.949	222	0.860	0.990
Radial	500–600	0.975	144	0.952	0.996

The SVM was considered fully trained at the approximate training set size at which the CV F_1_ score stopped improving—around 1,200–1,350 for the carotid and brachial but around 500–600 for the radial. The close agreement between the scores of the training and CV sets indicates the classifier did not overfit. The SVM achieved its highest CV F_1_ score on the radial data, second highest on the carotid data, and lowest on the brachial data, although all scores were over 94%. All arteries required a substantially smaller training set (<250) to hit a CV F_1_ score that was 97.5% of the final score, with the radial hitting this value with a training set size of 144—the smallest used.

For each artery, we have identified the precision for when the decision threshold was set to achieve 99% recall. The SVM performed best on the radial data with a precision of 95%, followed by the carotid (87%) and brachial (86%) data.

The ROC curve shows the true positive rate (sensitivity) against the false positive rate (1 − specificity). Sensitivity and specificity measure the ability of the SVM to correctly identify those with and without HF, respectively; therefore, the better the model performs, the closer its scores will be to the top left corner of the plot. This is quantified by the area under the curve; again, the SVM performed best on the radial data with a score of 0.996, followed by the carotid (0.994) and brachial (0.990) data.

#### Testing

The confusion matrices and summary statistics upon testing are given in Tables 7, 8: Table 7 for the default decision threshold and Table 8 for the 99% recall threshold. For the default threshold, the classifier performed best on the radial data with the scores of the other arteries close behind. For the 99% recall decision threshold, similar precisions were achieved to those given in Table 6, and here the SVM performed substantially better on the radial data with only 16 out of 400 subjects misclassified; there were 41 and 46 misclassifications out of 400 for the carotid and brachial data, respectively.

**TABLE 7:** Confusion matrices for each artery (top three) and summary statistics (bottom) for when the SVM was applied to the test set using the default decision threshold. HF = Heart failure.

**Common carotid**		**Actual**	
		**HF**	**No HF**
Predicted	HF	194	12
	No HF	7	187
**Brachial**		**Actual**	
		**HF**	**No HF**
Predicted	HF	187	11
	No HF	14	188
**Radial**		**Actual**	
		**HF**	**No HF**
Predicted	HF	197	7
	No HF	4	192
	**Precision**	**Recall**	**F**_**1**_ ** score**
Common Carotid	0.942	0.965	0.953
Brachial	0.944	0.930	0.937
Radial	0.965	0.980	0.973

**TABLE 8:** Confusion matrices for each artery (top three) and summary statistics (bottom) for when the SVM was applied to the test set using the 99% recall threshold. HF = Heart failure.

**Common carotid**		**Actual**	
		**HF**	**No HF**
Predicted	HF	201	41
	No HF	0	158
**Brachial**		**Actual**	
		**HF**	**No HF**
Predicted	HF	198	43
	No HF	3	156
**Radial**		**Actual**	
		**HF**	**No HF**
Predicted	HF	200	15
	No HF	1	184
	**Precision**	**Recall**	**F**_**1**_ ** score**
Common Carotid	0.831	1	0.907
Brachial	0.822	0.985	0.896
Radial	0.930	0.995	0.962

#### Errors

**FIGURE 7:**
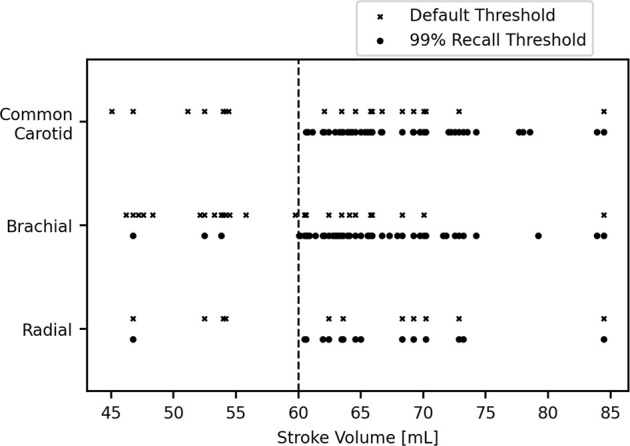
Stroke volumes of the misclassified subjects. The dashed line is the cut-off stroke volume.

The SVs of the misclassified test subjects are given in Figure 7.

For the default threshold, the majority of the SVs are close to the 60 ml cutoff, as expected, but several are situated around the 55 ml mark. The most extreme misclassifications either side were for a patient with an SV of 45.0 ml and a control with an SV of 84.5 ml with the former occurring for the brachial data and the latter misclassified for all datasets. There were multiple false negatives for those who had SVs less than 50 ml, indicating that this technique may miss several severe cases of impaired SV.

For the 99% recall threshold, most misclassifications are in the 60–75 ml range. There are three missed patients in total, with one being misclassified for both the brachial and the radial data. The most extreme misclassifications either side were for a patient with an SV of 46.7 ml and a control with an SV of 84.5 ml; the former occurred for both the brachial and radial and the latter for the brachial.

The percentages of severe misclassifications (patients who were classified as healthy despite having SV < 50 ml) were low for each artery and threshold: for the carotid, 0.5 and 0% were severely misclassified for the default and 99% recall thresholds, respectively; for the brachial, the same figures were 1.25 and 0.25%; for the radial, both were 0.25%.

## Discussion

Overall, the SVM achieved high precision and recall when using the default decision threshold for all arteries and maintained a high precision with the 99% recall threshold for the radial. We conclude that arterial WIA has significant potential to improve the HF diagnostic pipeline.

These results were achieved without separating the WI into intensities of forwards- and backwards-travelling waves, which eliminates experimental errors introduced by determining the PWV from the lnDU -loop. Additionally, stratifying the data by sex and age group did not improve the classification. Both of these results led to a simpler analysis without sacrificing performance. We may have neglected modelling criteria that would cause greater differences between the WIs of different sexes and age groups; nevertheless, even without such stratification, this study has already supported the ability of WIA to screen for HF.

In this study, the SVM performed best on the radial data, and this may in part be caused by differences in the RCs between arteries. In general, there was more stratification for the RC in the radial than for the other arteries; the radial was modelled as a terminal vessel, and increasing the peripheral resistance to keep pressures in the physiological range increased the size of the R wave relative to the S wave. The trend towards larger reflections was not observed in the brachial, probably as a result of wave trapping: the geometry of the arterial system is optimised to minimise reflection of forwards-travelling waves at bifurcations—the well-matched condition—but this necessarily results in poor matching for backwards-travelling waves, with a greater proportion of these being re-reflected (Parker, 2009). Hence, reflections generated in the periphery will be less apparent in the brachial than in the radial. (The radial artery performs well in other applications where wave properties are important—see Zheng et al., 2012.) This does not, however, explain why the trend towards greater reflection in HF patients occurs in the carotid too, which itself is one bifurcation removed from terminal vessels (the internal and external carotids).

For the default decision threshold, some subjects that had a severely impaired SV were misclassified as healthy, which is a significant issue. Altering the threshold to increase recall prevented the most egregious misclassifications, and this caught all patients in the carotid but not in the brachial or radial. Although there was some crossover in the misclassified patients between arteries, it may be the case that WI data from the carotid, brachial and radial could be used together to improve the results obtained here. Another issue, albeit one less serious, is the misidentification of healthy subjects as having an impaired SV: as noted above, these errors, which are small in number, would be corrected at the echocardiogram stage. Due to the inner complexity of the SVM, it is difficult to suggest why patients were misclassified; these errors warrant further investigation since the method may miss subjects that require immediate treatment.

An SVM classifier worked well for all three arteries examined in this study and gave particularly good results for the radial artery data. It is a versatile algorithm capable of identifying complex nonlinear patterns between metrics when classifying. SVMs are particularly effective in high dimensional spaces, but this was not advantageous here given the relatively low number of features. They are also well suited to small- to medium-sized datasets. Both of these advantages in part explain why SVMs have been successfully used in other biomedical applications: fields such as genomics are often characterised by high-dimensional data (Amaratunga and Cabrera, 2018), and sample sizes in typical clinical trials tend to be relatively small. One notable drawback of an SVM is the lack of interpretability: knowledge of the features that drive classification is important for developing a stronger understanding of heart failure itself and helpful for clinical adoption. Another drawback is that SVMs do not ordinarily provide a probabilistic estimate when classifying each subject; classification is given only in a binary format. Platt scaling is a method that can transform the outputs into a probabilistic model (Platt, 1999), but this comes with its own disadvantages.

Clinically, this method would require spatiotemporally coincident measurements of diameter and velocity with sufficient temporal resolution to resolve the waveforms accurately. MRI has previously been used to calculate WIs with a temporal resolution of 10 ms (Bhuva et al., 2020; Biglino et al., 2012; Li et al., 2010; Neumann et al., 2018; Schäfer et al., 2018), but it is currently unavailable in primary care or on the ward and is likely to remain so. The present study used a frequency equivalent to 1,000 frames per second, which is achievable with ultrafast ultrasound devices; previous studies have indeed used ultrasound to determine diameter and velocity using a combination of M-mode and Doppler (Feng and Khir, 2010), but the optimal beam angle for Doppler is orthogonal to that for M-mode, which introduces inaccuracies. Ultrasound imaging velocimetry is, however, feasible, for it can accurately determine the velocity waveform even in the presence of the high gradients observed in early systole, and wall-tracking algorithms can determine the diameter waveform with the necessary spatiotemporal coincidence. Real-time implementation of an ultrafast ultrasound method is also possible: scans can be acquired in a few seconds, beamformed in real-time with GPU accelerated computing (Hyun et al., 2019), and clutter-filtered in real-time with randomised singular value decomposition (Song et al., 2017). Subsequent processing to determine the wave intensity metrics is not computationally intensive, and results could again be available within seconds. There are, however, key challenges that must be addressed to permit successful clinical adoption of such a method, the most important of which is proving that it gives reliable, reproducible and accurate results when applied to real human data.

### Limitations

1. HF is a broad spectrum of disease that cannot be completely characterised by an impaired SV, although that can be a useful indicator.
2. In practice, not every HF patient will exhibit an impaired SV at rest, and WIA may only be able to identify such people under exertion.
3. The machine learning model used here was trained and tested on purely *in-silico* data, and strong performance on the generated datasets does not necessarily imply similar performance on actual patient data. However, this study was intended to propose methodology that could be applied in a real setting; the results provided here require corroboration by an experimental trial where measurement errors, amongst other things, could worsen the efficacy of the technique.
4. The only compensatory mechanism we have modelled is increased vascular resistance to maintain blood pressure. In reality, several neurohormonal compensatory mechanisms are involved in the compensatory response.
5. The population size used in this study was large when compared to that of a typical trial; however, the classifier could be trained to a reasonable standard with far fewer subjects. Furthermore, an alternative form of statistical analysis on a smaller sample may achieve similar success.

## Data Availability

The datasets presented in this study can be found in online repositories. The names of the repository/repositories and accession number(s) can be found below: https://github.com/ryanreavette/WaveIntensityData.
